# Supplementary material for: Fertilizer profitability for smallholder maize farmers in Tanzania: A spatially-explicit ex ante analysis
Source: PLoS One. 2020 Sep 18;15(9):e0239149. doi: 10.1371/journal.pone.0239149 (PMC7500610; doi:10.1371/journal.pone.0239149)

Supporting information for:

*Fertilizer profitability for smallholder maize farmers in Tanzania: A spatially-explicit ex ante analysis.*

Sebastian Palmas and Jordan Chamberlin

# Table A. Weather elements, soil properties and infrastructure variables used for the prediction of maize market price.

| **Raster** | **Short description** | **Derived from:** |
| --- | --- | --- |
| BIO1 | Mean annual temperature (°C * 10, 1979-2013) | [CHELSA](http://chelsa-climate.org/downloads/) |
| BIO7 | Mean annual temperature range (°C * 10, 1979-2013) | [CHELSA](http://chelsa-climate.org/downloads/) |
| BIO12 | Mean annual precipitation (mm/yr, 1979-2013) | [CHELSA](http://chelsa-climate.org/downloads/) |
| BIO15 | Mean rainfall seasonality (cv, 1979-2013) | [CHELSA](http://chelsa-climate.org/downloads/) |
| BPP15 | Predicted 2015 building presence probability | [Walsh, et al. (2019)](http://doi.org/10.17605/OSF.IO/J8Y3Z) |
| CEC | Predicted topsoil cation exchange capacity (cmol/kg) | [SoilGrids](https://soilgrids.org) |
| CPP15 | Predicted 2015 cropland presence probability | [Walsh, et al. (2019)](http://doi.org/10.17605/OSF.IO/J8Y3Z) |
| DCELL | Distance to Cell Tower or WiFi point (km) | [UnwiredLabs](https://unwiredlabs.com) |
| DFRES | Distance to forest reserves (km) | [Protected Planet](https://www.protectedplanet.net/) |
| DGRES | Distance to game reserves (km) | [Protected Planet](https://www.protectedplanet.net/) |
| DHRSL | Distance to High-Resolution Settlement Layer (km) | [CIESIN](https://ciesin.columbia.edu/data/hrsl/) |
| DNLT | Distance to BlackMarble night-lights (km) | [NASA](https://earthobservatory.nasa.gov/features/NightLights/page3.php) |
| DOR1 | Distance to main roads (km) | [Geofabrik](#https://www.geofabrik.de/data/download.html) |
| DOR2 | Distance to any known road (km) | [Geofabrik](#https://www.geofabrik.de/data/download.html) |
| DOWS | Distance to inland water bodies (km) | [SurfaceWater](#https://global-surface-water.appspot.com/) |
| DPARK | Distance to national parks & conservation areas (km) | [Protected Planet](https://www.protectedplanet.net/) |
| DPOP1 | Distance to city or town centers (km) | [Geofabrik](https://www.geofabrik.de) |
| DPOP2 | Distance to village or hamlet centers (km) | [Geofabrik](https://www.geofabrik.de) |
| EVI | Average enhanced vegetation index (2000-2016) | [Walsh, et al. (2019)](http://doi.org/10.17605/OSF.IO/J8Y3Z) |
| FIRE | Detected fire density (2001-2015) | [Walsh, et al. (2019)](http://doi.org/10.17605/OSF.IO/J8Y3Z) |
| GBD | Building footprint densities (2017) | [DigitalGlobe](https://explore.digitalglobe.com/Tanzania-Building-Footprints.html) |
| GCCP | USGS cropland extent prediction (2015) | [USGS](https://croplands.org/app/map?lat=0.17578&lng=-0.17578125&zoom=2) |
| GFPL | Global floodplain extent (2017) | [USGS](https://www.usgs.gov/centers/eros/science/usgs-eros-archive-landsat-landsat-level-3-dynamic-surface-water-extent-dswe?qt-science_center_objects=0#qt-science_center_objects) |
| LCB | Bare /sparse vegetation cover fraction (2015) | [Copernicus](http://lcviewer.vito.be/) |
| LCC | Cropland cover fraction (2015) | [Copernicus](http://lcviewer.vito.be/) |
| LCS | Shrubland cover fraction (2015) | [Copernicus](http://lcviewer.vito.be/) |
| LCT | Forest cover fraction (2015) | [Copernicus](http://lcviewer.vito.be/) |
| LCU | Built-up cover fraction (2015) | [Copernicus](http://lcviewer.vito.be/) |
| LSTD | Average day-time land surface temp. (°C, 2001-2017) | [Walsh, et al. (2019)](http://doi.org/10.17605/OSF.IO/J8Y3Z) |
| LSTN | Average night-time land surface temp. (°C, 2001-2017) | [Walsh, et al. (2019)](http://doi.org/10.17605/OSF.IO/J8Y3Z) |
| MB1 | Average MOD13Q1 band 1 reflectance (2001-2016) | [Walsh, et al. (2019)](http://doi.org/10.17605/OSF.IO/J8Y3Z) |
| MB2 | Average MOD13Q1 band 2 reflectance (2001-2016) | [Walsh, et al. (2019)](http://doi.org/10.17605/OSF.IO/J8Y3Z) |
| MB3 | Average MOD13Q1 band 3 reflectance (2001-2016) | [Walsh, et al. (2019)](http://doi.org/10.17605/OSF.IO/J8Y3Z) |
| MB7 | Average MOD13Q1 band 7 reflectance (2001-2016) | [Walsh, et al. (2019)](http://doi.org/10.17605/OSF.IO/J8Y3Z) |
| MDEM | Elevation above mean sea level (m) | [MERITDEM](http://hydro.iis.u-tokyo.ac.jp/~yamadai/MERIT_DEM/) |
| NPPA | Average annual net primary productivity (kg/m^2^, 2000-2015) | [Walsh, et al. (2019)](http://doi.org/10.17605/OSF.IO/J8Y3Z) |
| NPPS | Variance annual net primary productivity (2000-2015) | [Walsh, et al. (2019)](http://doi.org/10.17605/OSF.IO/J8Y3Z) |
| PARA | Average fAPAR (2000-2017) | [Walsh, et al. (2019)](http://doi.org/10.17605/OSF.IO/J8Y3Z) |
| PARV | Variance fAPAR (2000-2017) | [Walsh, et al. (2019)](http://doi.org/10.17605/OSF.IO/J8Y3Z) |
| PH | Predicted topsoil pH | [SoilGrids](https://soilgrids.org) |
| S1VV | Average Sentinel 1 VV radar backscatter (2016) | [Walsh, et al. (2019)](http://doi.org/10.17605/OSF.IO/J8Y3Z) |
| S2B11 | Average Sentinel 2 SWIR1 reflectance (2016-2017) | [Walsh, et al. (2019)](http://doi.org/10.17605/OSF.IO/J8Y3Z) |
| S2B12 | Average Sentinel 2 SWIR2 reflectance (2016-2017) | [Walsh, et al. (2019)](http://doi.org/10.17605/OSF.IO/J8Y3Z) |
| SLOPE | Slope (%) | [MERITDEM](http://hydro.iis.u-tokyo.ac.jp/~yamadai/MERIT_DEM/) |
| SND | Predicted topsoil sand content (%) | [SoilGrids](https://soilgrids.org) |
| SOC | Predicted topsoil organic carbon content (g/kg) | [SoilGrids](https://soilgrids.org) |
| TIM | Topographic index map | [MERITDEM](http://hydro.iis.u-tokyo.ac.jp/~yamadai/MERIT_DEM/) |
| WPOP | Population density prediction (2015) | [WorldPop](http://www.worldpop.org) |
| WPP17 | Population density prediction (2015) | [WorldPop](http://www.worldpop.org) |

# Fig A. Summary of variable importance in the yield production model from a Random Forest regression


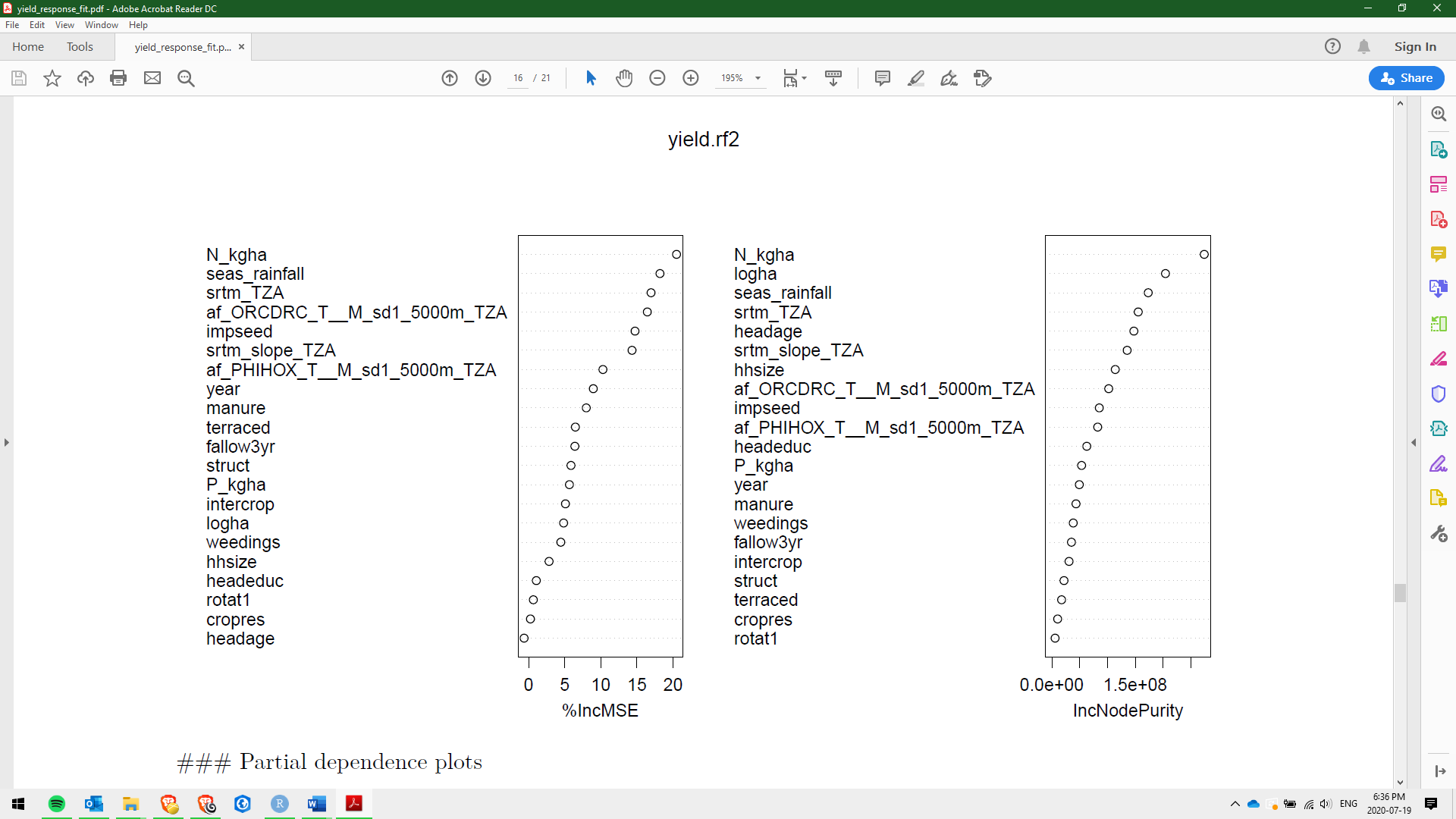


| **Variable name** | **Variable** |
| --- | --- |
| N_kgha | Applied Nitrogen (kg/ha) |
| P_kgha | Applied Phosphorus (kg/ha) |
| seas_rainfall | Seasonal rainfall (Dec-May) |
| srtm_TZA | Elevation (m) |
| af_ORCDRC_T__M_sd1_5000m_TZA | Soil organic carbon (kg/ha) |
| srtm_slope_TZA | Slope (°) |
| Impseed | Use of improved seeds (yes = 1) |
| Year | Year of survey |
| af_PHIHOX_T__M_sd1_5000m_TZA | Soil pH |
| struct | Erosion control structure (yes = 1) |
| manure | Use of manure (yes = 1) |
| fallow3yr | Field in fallow in the last 3 years (yes = 1) |
| intercrop | Intercrop (yes = 1) |
| logha | Area in hectares of focal plot (log) |
| terraced | Terraced field (yes = 1) |
| hhsize | Household size |
| weedings | Number of weedings |
| headeduc | Years of education of head of households |
| rotat1 | Crop rotation (yes = 1) |
| cropres | Use of crop residue (yes = 1) |
| headage | Age of head of household (year) |

# Fig B. Fertilizer price model. (A) Price model defining the rate at which delivered fertilizer prices increase with distance from markets. (B) Resulting nitrogen fertilizer price distribution in Tanzania.


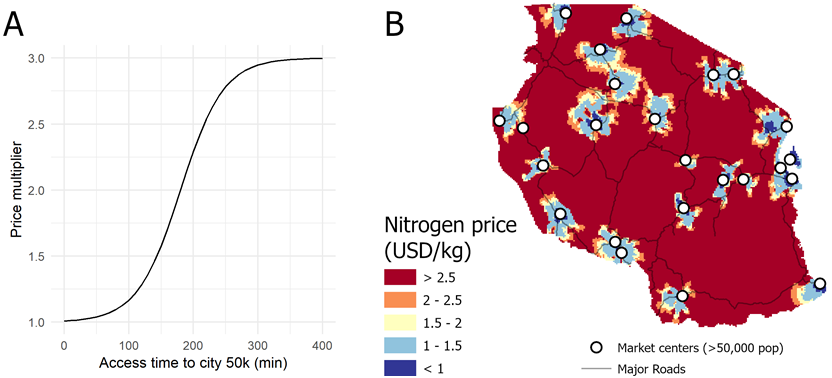


# Fig C. Map of simulation results by scenario. Predicted yields and yield gains from ZERO. Predicted net revenue and net revenue gains from ZERO. Base maps of Tanzanian administrative regions were obtained from the GADM database of Global Administrative Areas (http://www.gadm.org/, last accessed October 2019).


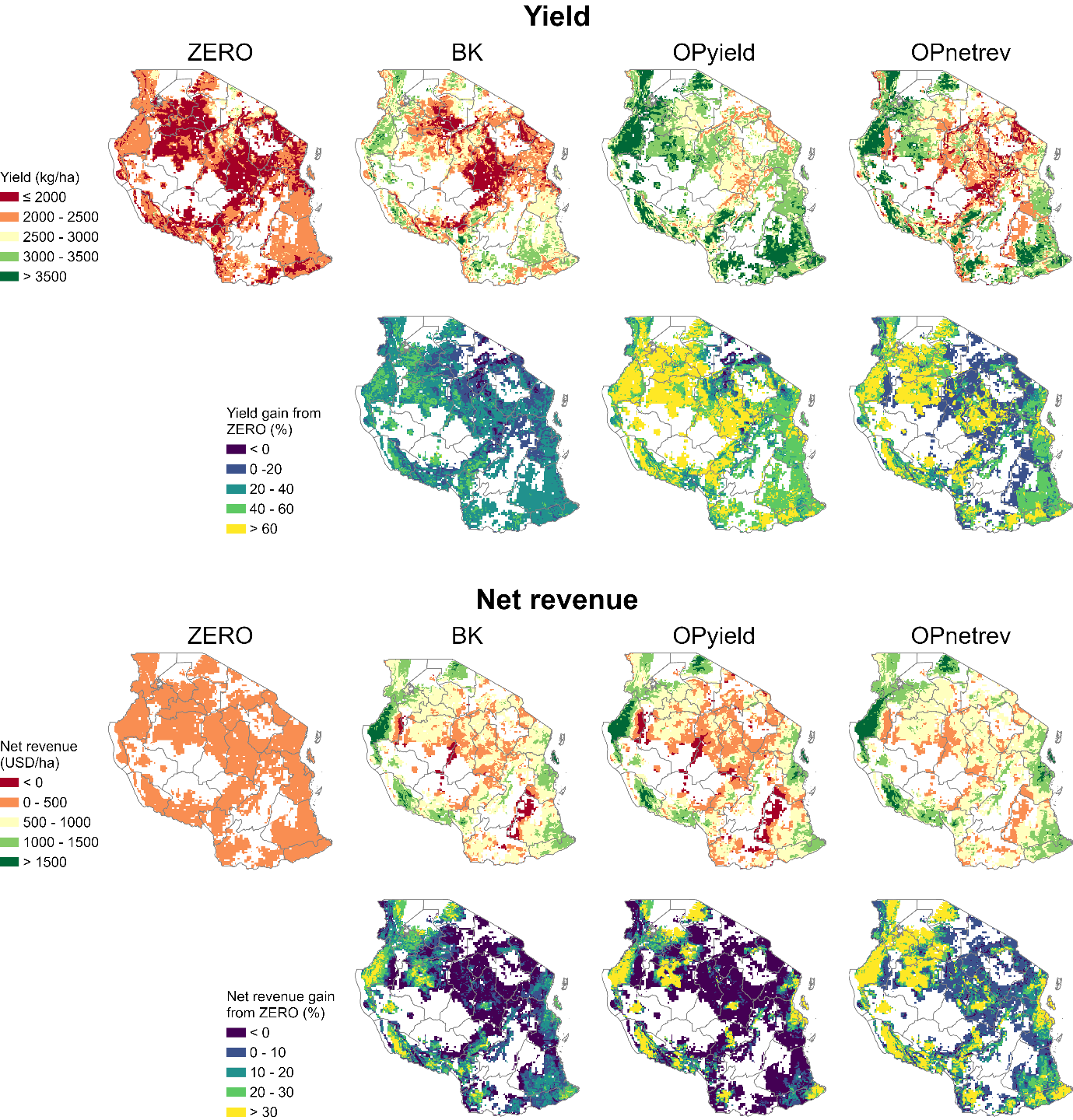

Supplement: S1 Text — (DOCX) [file pone.0239149.s001.docx]
